# Dr. Wright’s Address to Graduating Class O. C. D. S.

**Published:** 1852-04

**Authors:** Wm. M. Wright

**Affiliations:** Pittsburgh, Pa.


### DENTAL REGISTER OF THE WEST.

Vol. V. APRIL, 1852. No. 3.

### Original Communications.

### ADDRESS TO THE GRADUATING CLASS

*Of the Ohio College of Dental Surgery, delivered 20th February*, 1852,

BY WM. M. WRIGHT, M. D., OF PITTSBURGH, PA.

Young Gentlemen: You have now arrrived at the period to which you have been looking forward as the termination of your pupilage. This is in many respects a most interesting and important period of your lives. You are about to dissolve present relations, and to form others new and untried; having finished a course of education preparatory to the practice of an important profession, your minds are perhaps alternately expanding with high anticipations of success in your allotted sphere of life, or depressed by the apprehension of disappointment, as hope or fear

* By request of the graduating class of the Ohio College of Dental Surgery, we publish the address of Dr. Wright, who by request of the class has furnished us with the copy. We feel assured the profession will respond to the sentiment of the address, and agree with those who heard it delivered in a very happy and felicitous manner, that it will well repay a perusal. The commencement exercises passed off very pleasantly, and the happy coincident of sentiment in the very admirable address of Rev. B. P. Aydelott, D. D., President of the Board of Trustees, and that of Dr. Wright, was truly admirable, and added, as we think, interest to the occasion.

for the moment preponderates—you stand upon the line which is to separate you from your instructors—those who with assiduous and generous care, have nurtured to conscious strength your immature faculties, have conducted you through the incipient stages of learning, furnishing your minds with an amount of useful and practical knowledge, which qualifies you now to assume a station for yourselves wherein to develope and employ your powers as gifted by nature and improved by their full cultivation, you are now to lose the staff on which you have rested for support—and with the world before you, stand ready to encounter its vicissitudes, to grapple with its difficulties, relying on yourselves alone for success, when friends and aid may not be present.

In every important enterprise much depends on a favorable commencement—moral no less than physical events are connected by the relations of cause and effect. A single misstep at the outset in entering upon active life, by its influence on the mind and on outward circumstances may operate to cloud totally and forever the fairest prospects. The future is now before you—what a word of startling moment!—but it has been beautifully said, "The veil which covers the face of futurity is woven by the hand of mercy."

Being for the occasion permitted to assume a position authorizing me to act in the relation of a friend, and to address you as young men about to take your place in the great society of man, my wish is to make a few *practical observations* of a very *general character*, that may have a bearing on your future welfare and success in life.

You form part of a numerous and respectable class of men, on whom the hopes of our common humanity rest. We sustain its honor and interests in an important department of science. I trust you will bring to the aid of your profession, and of society in every relation you may sustain to it, no small increase of faithful service, guided by talents cultivated with care, and exerted with laudible zeal.

You have surveyed the foundations of that particular science which it will be your business to cultivate; enriched your understandings with the principles of general and professional knowledge, and by discipline and exercise have acquired a vigor and activity of mind the value of which is not to be estimated by wealth. But I would here caution you against the supposition that your mental stores and resources are complete; that the business of education is finished. Such an opinion would be in the highest degree dangerous and fatal to your future prospects. Would you occupy a high position among professional and literary men—would you attain to the perfection of your faculties, accomplish the greatest amount of good to yourselves and others, and reach the highest destination to which it is the will of Providence you shall aspire, think not to lay aside the character of student when you assume that of practitioner—you must cultivate the power and habit of systematic, close, laborious and patient study and observation—this habit must go with you through life, and be brought to bear on everything presented to your consideration, and must be extended not merely to the specialties of our profession, but to all the sister and kindred branches of knowledge.

Knowledge, unless frequently renewed, regulated and enlarged by study, gradually becomes less; present ideas escape through the weakness of memory, and the mind by degrees relapses into its former ignorance, or retains only those ideas which are kept alive by the daily routine of professional duties. As to the means necessary to preserve their health and vigor, there is an obvious resemblance of the mental faculties to those of the body. Were you from this hour to cease exerting your bodily powers, how long would they retain their present strength and activity? Not many weeks. The physical constitution would very soon become enfeebled, the buoyancy and enjoyments of health would be rapidly exchanged for the languor and pains of disease. Effects entirely analagous and quite as observable as these, follow mental inaction. The diseases of the mind are not generally perceived and acknowledged, because so general, as to be mistaken for a natural state; but they are not on this account less real. It is from the enfeebling effects of mental inactivity, that a large portion of mankind are utterly incapable of judging correctly on many subjects of vital importance to themselves. To this, as one of its principal sources, we may trace the charlatanary which disgraces, and hangs as a vampire, sucking the life-blood from the learned professions.

It is this chiefly which has converted into dentists hundreds of men, who have laid down the implements of their trade, and taken up those of our profession, without education or preparatory training, and given them emoluments which should have been devoted to sustain and encourage professional merit. would men give themselves the trouble to think, they could not be so easily imposed on; but so far as men neglect to exercise their own intellects in obtaining certain knowledge, on which to decide the great questions of truth and duty, they will necessarily follow the dictates of others—no matter how contrary to their own interests. If then you would enjoy intellectual freedom, you must not calculate on a life of mental indolence; you must study books and nature, read extensively and think deeply, patiently, with an unprejudiced and discriminating judgment; learn to trust your own faculties. Think not yon can go no farther than you are led and supported by others. Remember those whose opinions have governed the world were once without full knowledge or judgment. A sincere love of truth, with patient, systematic industry in the pursuit of it, will enable you ou all important subjects connected with your profession and calling in life, to judge as correctly as human frailty will allow. Scarcely anything tends so much to debase the mind and cripple its energies as copying others. It entirely destroys expansion and froedom of thought. The mind creeps along the shore of other men's opinions, following their zig-zag course, in continual danger of falling on shoals and concealed rocks of error, instead of launching out boldly into the great ocean of truth, guided by the compass of rational investigation.

There are two properties of mind of great importance in literary and scientific pursuits. The first is humility, founded on a conviction of our natural ignorance. The limited extent of the human faculties, and the almost boundless limits even of attainable knowledge. It is this feeling which explains the seeming paradox, that a man is only then becoming truly wise when he has discovered that he knows nothing. Sir Isaac Newton, with his wealth of learning, said he felt like a child picking up pebbles on the shore of the great ocean of knowledge.

It is opposed to what Mr. Locke calls presumption, an exaggerated, false estimate of our powers, leading us to neglect their cultivation, and filling the head with vain, ignorant conceits. But while yon avoid such a partiality to your parts or attainments, as to suppose yourselves prepared for every occasion without the labor of deep reflection and constant study, practice and observation, you should avoid the opposite extreme of falling into despondency, of suffering the mind to be depressed and its energies paralyzed by imagining yourselves unequal to any great undertaking. Do not permit yourselves to be turned aside or cast down, on account of failing many times to accomplish an end. "If a spider break his thread a hundred times, a hundred times will he mend it"—make up your minds to do a thing, and you will do it. "There is no difficulty to him who wills."

> "The wise and active conquer difficulties
> By daring to attempt them: sloth and folly
> Shiver and sink at sights of toil and hazard,
> And make the impossibility they fear."

It is the observation of a great philosopher that no one knows the extent of his powers until he has tried them, and that generally on trial, they will be found much greater than supposed. By steady, persevering, systematic exertion, you may acquire a power of mind and attain a professional standing, surprising even to yourselves, and realizing your highest dreams of ambition and professional aspiration.

If, in the outset of professional life, contemplating the obstacles you will need to surmount in your way to fortune or fame—if, in reflecting on those who are loitering at the threshold of the dental profession, upon which they have entered many years ago, or have fallen by the way, for want of strength to reach the goal, or judgment to direct them to the path which leads to it, should you doubt your capacity for self-dependence, and fear to take the first step, reflection will scatter these vapors, clear the intellectual vision, and summon resolution to the aid of the mind. You will find encouragement in the example of those men—many of whom still live to shed around dental science the lustre of their great acquirements and world-wide fame, whose names are identified with its history, but when in entering the profession, from the infancy of the science, the paucity of books on the subject, and the difficulty of obtaining these, were obliged to surmount obstacles, which happily you will not have to encounter. With stout hearts, dexterous hands, and clear heads, they wrought their way to ultimate distinguished success. Stimulated by such worthy examples, be like them, determined to reach your object. Let it be your ambition, as it was theirs, to fill to the brim the full measure of your position; let your steps be guided by a laudible pride to add something to the results of their labors, which shall benefit mankind beyond the brief period of individual existence. With advantages which they had not, follow their example by making as good use of the means which you possess, as they did of what was in their power, and you will find, in the swelling resources of your profession, abundant room and material on which to display your talents, apply your knowledge and industry, so as to meet the highest expectations of Alma Mater, of all interested in your welfare, or the advancement of useful knowledge, and the melioration of our kind.

Labor to improve and complete what the noble men in this department of professional science have so recently and gloriously begun. Something can be done by each of you in your respective fields of labor, and though it be not great, your separate productions brought together, might in the aggregate, form a large addition to the common stock, and make the profession greatly your debtors.

There is no idle way of becoming great or illustrious in any science or art—labor is the price set upon everything valuable, and be assured, no man, let his genius be what it will, ever rose to eminence but by unwearied devotion. Dryden said, "There was no royal road to knowledge:" let me assure you, young gentlemen, there is no railroad to perfection in art.

"The clouds may drop down titles and estates
Wealth may seek us—but wisdom must be sought."

And he who starts into being one thrill of vital thought—who adds an idea to the previous stock of human knowledge, is a benefactor of his race, and deserves to be held in everlasting remembrance.

A single idea, indeed, is often no inappreciable quantity. In some instances, in its immediate results, it is a vast augmentation of knowledge, extending the intellectual field of hitherto unexplored territory, revealing mysteries kept secret from the foundation of the world. What was Lord Bacon's great addition to science, which has entitled him to the appellation of father of modern science ? The single idea that the whole of natural philosophy consists of a series of inductive generalization, commencing with the minutest particulars, and carried up to universal laws and axioms. And what was Newton's contribution to knowledge, which has given immortality to his name, and to the world the divine science of astronomy ? The single idea that all matter gravitates to a common centre. The least contribution you may be able to make to the stock of professional knowledge, though it be but a single new fact, may in its results, when combined with others, prove vastly important. In Holy Writ we are taught to look to small things as the origin of the greatest; even the kingdom of heaven is compared to a grain of mustard seed, not to an acorn, or nut, but to the least of seeds, yet containing a latent principle capable of sudden and wonderful expansion, and may not irreverantly be taken to teach us the importance to the advancement of science, of isolated facts, however small or trivial they may appear, at first view. The old maxim also applies here, "Take care of the penny, and the pound will take care of itself"—take care of the thousand little facts, furnished by every day's experience and observation, the induction of the general principle or law of nature under which they have been developed, will necessarily follow. No one knows till he tries, what will be the result of the close observation of nature, as brought to view through his particular calling in life; her works and mode of working, of repeated and varied experiments, of associating facts with general principles, and of contemplating both by the light thus mutually reflected. You may fail in the immediate object of your experiment, yet develope something else connected with the profession, of equal or greater importance, as the alchymists who could not turn other metals into gold, nevertheless were led to an accumulation of facts, and to disclosures of nature incomparably more important to mankind. Such labors are sure of recompense, both in the consciousness of doing good, and in the ultimate reward of mankind. Imposters may have their day, and public justice may be slow in discriminating those who are deserving, but time, which tries men and their works, is certain in the end to separate the precious from the vile, and entwine its laurels around the brow of true merit. And honors thus won are never lost. This is especially true of honors bestowed on those who have benefitted mankind by improvements in the healing and the useful arts Unlike the statesman and politician, who find the "stairs to honor steep, the standing slippery, the regress a downfall," these need no support in patronage to give them firm footing. Their standing is impregnable, there is no downfall or decay to their honors, no farewell to their greatness, which will be as permanent and universal as the influence of their labors, and this will be commensurate with time and space.

As a means, young gentlemen, of individual improvement, and of usefulness to the profession, allow me to urge upon your attention, the frequent use of the pen, and that you labor to acquire a clear and forcible style of writing. Without this, talent and learning, though of transcendant character, will exert but a limited influence. Sentiments uttered by the living voice, may perhaps influence a few thousands—uttered through written language, they may influence millions, may awaken the dormant energies of a nation, or arouse the whole civilized world, and be transmitted to future generations. Through this medium the wisdom and virtue of past ages stlll live to instruct and bless mankind. But it is the press which in the present age gives to written language its amazing power. The press now governs the world. Empires rise and fall by its influence.

Institutions flourish or decay as they are supported or opposed by it. Science is endebted to it for its onward march. It is the mighty engine, which operating with silent but resistless energy, through the whole social frame, is removing its chronic maladies and restoring it to health. A vast popular government like ours, founded on equality of rights, admitting universal suffrage and dependent on the will of the masses, could not exist without the press, to diffuse knowledge and morality among the people. It is the channel through which the fountains of knowledge burst, and send forth their streams to gladden every part of the earth. If then, young gentlemen, you would ally yourselves to any of the great benevolent enterprizes of the day—and they are numerous enough to gratify the most fastidious ambition—if, especially, you would contribute to the triumphs of our professional knowledge, by communicating to the world the results of your own reflections, observations and experience, you must be able to wield the power of the press. And let not one of you imagine himself incapable of becoming a correct and able writer. Perhaps no art depends more on practice and habit. Plain common sense is the only requisite necessary to become a forcible and convincing writer. Industry and perseverance will accomplish the rest. Contemplating the astonishing feats of body performed by those who exhibit themseives for pay, some of which would be incredible were they not so well attested, we would naturally conclude that these men differ from other men in their physical constitution. The truth is they are but ordinary men, and have acquired this astonishing skill in their arts by practice and discipline. Thus you see the influence of habit on the body; and is the mind less susceptible of improvement? An equal degree of labor and pains in acquiring the power of writing, would in most cases secure a high degree of success. I have introduced this topic here, and thus urge it upon your consideration, because I believe the present position of dental science before the world demands clear-headed, intelligent, practical aud forcible thinkers and writers. If we are true to its interests and feel determined that it shall not be subsidiary to any of the learned professions—that it shall not be tacked on as an accommodation car to take up those who have failed to meet the requirements of that profession to which they have directed their time and energy. If we feel determined that it shall be placed, where from its high utility and great worth it deserves to stand, among the foremost and best of the useful and learned professions, we must rally around our dental colleges, and support our dental literature.

Closely connected with the subject of writing, is the systematic arrangement and use of time. To become writers, to attain to eminence in your profession, in short, to accomplish any great object of utility to yourselves or others, time must be diligently employed—time and labor are the price which Providence exacts of us for every blessing. There is a class who are forever complaining of the want of time for their duties, who yet spend hours in idleness, frivolous employment, or worse. The want generally is not time, but its systematic improvement. This is the true reason why some men have accomplished such wonders that to those of less orderly and diligent habits they seem to have possessed superhuman powers, at an early period of life perhaps have grasped almost universal knowledge, or in the midst of professional engagements, which seemed to deny them the opportunity of retirement, have become the most voluminous writers of their age. But you will say these men were highly gifted by nature, possessed of powerful talents, and therefore not to be presented as models for ordinary men. True, some of them were men of strong understanding; but who that has observation, does not almost daily meet with such in the humblest walks of life? Their great talents will not account for their success. Had they given themselves up to indolence, they had been but common men. It was diligence, and perseverance in the use of time which wrought such wonders, and carried them to that sublime elevation, whence they were conspicuous, and objects of admiration to the whole world.

Finally, gentlemen, you are now prepared for the race which is before you. May it be long and brilliant. You have chosen your ground, and have arrived at a point where you see the direction of your course. In connection with the honored institution you are about to leave, you have acquired an amount of knowledge, which not only secures you against the painful apprehension of failure, but prepares you for distinguished success. Though in consequence of this course of preparatory training, you enter later on the practical duties of the profession, both yourselves and the public will thereby be infinitely the gainers. Though time has been consumed, it has in return conferred on you its own priceless value, in those superior qualifications with which you now enter upon the world, to act your parts, we trust with distinguished honor to yourselves, and advantage to others. We do not say that such a course of public instruction is absolutely *indispensable* to professional success, or even eminence. Such a declaration would be unpardonably invidious, as well as untrue, contradicted as it is by many prominent examples to the contrary, both among the living and the dead. But it should not be forgotten, that those who have succeded without such preliminary aid, have done so, not *because* of their disadvantages in this respect, but in *spite of them.* If they did not avail themselves of the advantages of such an institution as this, it was not that they considered them unnecessary, or unimportant, but owing to the misfortune of their own circumstances, or of the times, which rendered them impracticable. And most deeply do many of them regret that they could not have enjoyed the opportunities of information and regular training in all the branches of dental surgery, which are now so abundant, and placed within the reach of every young man aspiring to an honorable place in the profession. That such advantages were not enjoyed by the older practitioners of the country, is not remarkable when it is reflected that their own experience goes back to the infancy of the science. You, gentlemen, commence your professional career under brighter and more favorable auspices. When dental science has reached its youthful vigor, and is fast ripening to maturity, may your labors, combined with others who are entering upon the stage along with you, do as much to advance the profession, to extend its principles, improve its mechanical execution, and multiply its honors, as the labors of the generation which is passing away.

It is the duty of every member of society, while laboring for his own benefit, to endeavor in some way to be useful to others. And I congratulate you, gentlemen, on the prospect of a calling in which there is no conflict of these interests, but which in a high degree secures both. Human happiness consists not in the acquisition of wealth or fame, or the amount of either. The desire of accumulating beyond the means of rational enjoyment for the present, or of wise provision for the future, is never satisfied. The man who has hoarded millions, wants something more; however large the useless heap, he wishes still to add to it, and this insatiable desire will never allow him to enjoy with content what he has. Ask the man of power if he is satisfied with the degree of influence which the constitution and laws of his country have confered on him, and he will give you to understand that in the distribution of the honors and powers of government, others have received too much, he too little. There could not be a greater error than to associate the idea of supreme felicity with the trappings of wealth and power, or with the pomp and parade of elevated stations. The true secret of happiness here lies in the placing the desires on objects depending more on ourselves than on the vicissitudes of fortune—objects easy of attainment, and that expose us as little as possible to the miseries of anxious solicitude and disappointment. Such it is believed, are the pleasures of professional and general literature, and the exercise of the benevolent affections in a life of usefulness.

After the very satisfactory manner you acquitted yourselves before the examining committee—alike creditable to yourselves and the learned gentlemen who have been lecturing to you during the session which has now closed. I think it would be out of place as well as uncalled for, if I should take up your time with the specialties of our profession.

In conclusion, allow me to say that professional honor and individual happiness, are inseparably connected with a virtuous life. Possessing real rectitude, "governed always by motives of duty, by sentiments of justice and benevolence, we need no effort to *appear happy*, we are so in truth, without which there is no true lasting enjoyment." Be patient in the pursuit of business—bide your time—you must succeed.

There is too much disposition in this world to arrive at the completion of certain matters, not having the patience to move slowly, surely and certainly. We are for planting full *grown trees* even if they should but flourish for a time, and then wither and leave a "blasted trunk remain." I would advise you first to prepare the ground, then plant the seed and wait patiently for the germination of the seed. Then anxiously look for the tiny green blade as it makes its way through its mother earth—and then cultivate it with care until it becomes the thrify twig—then the sturdy sapling—then the full-grown tree, large and umbrageous—protecting you alike from the scorching sun-time of life, as well as the winds of winter—gathering from its lofty boughs, rich, ripe fruit that cluster upon its branches.

At the close of Dr. Wright's address, Dr. Herring, one of the graduates, replied as follows:

Respected Sir: Permit me, in behalf of my fellow graduates of the Ohio College of Dental Surgery, to express to you our sincere thanks for the interest you have manifested in our future welfare. We hope ever to profit by the good counsel you have given, which we feel assured will lead us, if not to professional fame, to a useful and happy life. In return, permit us to hope that Heaven's brightest smiles may rest upon your future.

Gentlemen of the Faculty: To you we should fail to express the language of our hearts. We can only say, that for your generous treatment to us, and assiduous attention to your duties as teachers during the session now brought to a close, we shall ever feel deeply grateful.

Should we in future govern our actions by the instructions you have given us this winter, we feel that success is almost certain.

Permit us, then, gentlemen, to assure you that in the discharge of your duties—which have not been without some sacrifice—you have rendered the most peıfect satisfaction.

The high honors you have this evening conferred upon us, we hope ever to so maintain, that the profession will view with pride our Alma Mater, and the diplomas this evening received as evidenee of our attainments, may never be dishonored.

This is considered an age of progress, and perhaps no department of science shows more evidence of this than Dental Surgery.

The past twenty years records much which is new in dental practice, and can we not now regard perfection as nearer attained than ever before. We are led to believe that the course of our science is still onward and upward, each year developing new attainments, and we shall still look to this institution of our choice, and you, gentlemen of the Faculty, for much which shall give success to this onward progress.

In conclusion, we tender you our grateful acknowledgments, and bid you an affectionate farewell.

